# Curing conditions to inactivate *Trichinella spiralis* muscle larvae in ready-to-eat pork sausage

**DOI:** 10.1016/j.fawpar.2017.06.001

**Published:** 2017-06-23

**Authors:** D.E. Hill, J. Luchansky, A. Porto-Fett, H.R. Gamble, V.M. Fournet, D.S. Hawkins-Cooper, A.A. Gajadhar, R. Holley, V.K. Juneja, J.P. Dubey

**Affiliations:** a USDA, ARS, NEA, Animal Parasitic Diseases Laboratory, BARC-East, Bldgs. 1001 & 307-C, Beltsville, MD 20705, United States; b USDA, ARS, NEA, Food Safety and Intervention Technologies, 600 E. Mermaid Ln. ERRC, Wyndmoor, PA 19038-8598, United States; c National Academy of Sciences, 500 Fifth Street NW, Washington, DC 20001, United States; d University of Saskatchewan, Department of Veterinary Microbiology, 52 Campus Drive, Saskatoon, SK S7N5B4, Canada; e Faculty of Agricultural and Food Sciences, Room 250 Ellis Building, 13 Freedman Crescent University of Manitoba, Winnipeg, MB R3T 2N2, Canada; f USDA, ARS, Northeast Area, Animal Parasitic Diseases Laboratory, Center Road, Bldg 307-C, Rm 134, BARC-East, Beltsville, MD 20705, United States

## Abstract

Curing processes are one method by which pork products, which are considered ready to eat (RTE) and have not been otherwise tested or treated, can be rendered safe from risk for exposure to *Trichinella* muscle larvae (ML). Curing processes in the U.S. currently require individual validation of methods to demonstrate inactivation of *Trichinella*. This is a major undertaking for each process; currently no model of meat chemistry exists that can be correlated with inactivation of *Trichinella*. Given the potential for new RTE products (e.g., lower salt), the availability of a wider range of tested methods for inactivation of *Trichinella* in pork would be of substantial value to the industry. In this study, five variables were tested – salt/brine concentration, water activity (a_w_), pH, temperature, and time, using low and high endpoints for common curing treatments for dry cured pork sausage. The data demonstrated that NaCl concentrations above 1.3%, in combination with fermentation to pH 5.2 or below, resulted in inactivation of > 96% of *Trichinella* ML in stuffed sausages within 24–28 h. All ML were inactivated by 7–10 days post-stuffing. These curing processes reliably predict inactivation of *Trichinella spiralis*, and can be used within the defined upper and lower endpoint parameters to reduce or eliminate the need for individual product validation.

## 1 Introduction

Meat curing is a preservation process that is widely used in the pork industry. Curing processes are used to produce certain product characteristics, including increased shelf life and palatability/taste. While product safety, relative to microbial contamination, is addressed in Hazard Analysis and Critical Control Point (HACCP) plans, following requirements such as those of the United States Department of Agriculture (USDA), Food Safety and Inspection Service (FSIS) guidelines on fermented products, (United States Department of Agriculture, 2005), there are no general control guidelines for inactivation of parasites, such as *T. spiralis,* that may reside within pork meat. There are many processed pork products that rely on a form of curing (pepperoni, salami, and prosciutto); the USDA currently requires that cured pork products are prepared with pork that has tested negative for *Trichinella* at slaughter (adding a premium to the cost of raw product), or be prepared by one of the curing processes that has been validated to inactivate *Trichinella*. All validated and approved curing processes for inactivation of *Trichinella* are listed in the U.S. Code of Federal Regulations – 9 CFR 318.10 (U.S. Federal Register, 2012). These validation studies are difficult and expensive for producers to perform, and only seven approved methods are currently available. Additionally, there are no models that can be used to extrapolate the parameters of meat chemistry which result in inactivation of *Trichinella*; prediction of inactivation of *Trichinella* in pork meat cannot be accomplished with existing models for other pathogens. The prescriptive methods contained in 9 CFR 318.10 are proposed for elimination in favor of a HACCP approach to product safety in which producers are required to determine if *Trichinella* is a risk reasonably likely to occur in their product, and if so, they must take action to inactivate or eliminate the risk. Curing is one of the available inactivation options (Lin et al., 1990a, Lin et al., 1990b, Gamble et al., 1997, Pozio et al., 2006, Hill et al., 2009, Porto-Fett et al., 2010), but due to a lack of understanding of the parameters for inactivation, may only be applied using specific validated methods. The objective of this study was to develop de novo inactivation data for pork curing processes which could be used to determine the effect of dry curing treatments on *Trichinella*, based on meat chemistry of the final cured product. The study considered five common measurements monitored during curing – salt/brine concentration, a_w_, pH, temperature, and time. Using the newly developed inactivation data, existing and newly developed curing process that fall within the tested parameters can be assessed for risk mitigation with respect to *Trichinella* based on the meat chemistry of the final product. These data provide a baseline for the ultimate goal of developing a searchable *Trichinella* inactivation model which can be used to populate the USDA, ARS Pathogen Modeling Program (PMP; PMP is produced at the USDA-ARS Eastern Regional Research Center (ERRC) in Wyndmoor, Pennsylvania, download at https://pmp.errc.ars.usda.gov/PMPDownload/PMP80Setup.exe (stand alone version 8) or https://pmp.errc.ars.usda.gov (Online version)) and used by producers and regulators to assess the safety of new curing processes without the need for individual process validation.

## 2 Materials and methods

Time periods for analysis were developed in a pilot study, in which non–infected meat was fabricated as described below and samples were taken for meat chemistry on a daily basis. Based on the speed of fermentation, time periods for sampling infected meat were determined, and the process adjusted to meet the criteria required for typical commercially prepared product. For experimental data collection, a total of 30 mixed breed pigs, 10–12 weeks of age each, were infected per os with the Beltsville strain of *T. spiralis* first stage muscle larvae (ML) at 10,000 ML per pig. *Trichinella* ML were maintained in rats, and passed through pigs once per year. Pigs were housed at the USDA's Beltsville Agricultural Research Center, and grown to near market size (67 to 75 kg). Infected pigs were housed in separate pens and quarantined from non-infected pigs in accordance with the Animal Welfare Act, Guide for the care and use of laboratory animals, (2011) (8th Edition) and with the approval of the USDA/ARS Beltsville Area Institutional Animal Care and Use Committee (BAACUC Approval # 15-014). After 60 days, infected pigs were humanely euthanized by electric stunning followed by exsanguination. The triceps, picnics, hams, neck, rump, and loins were collected and hand trimmed of excess fat and connective tissue to yield a total of 23 to 30 kg of meat from each animal. Back fat was also collected from each animal and supplemented with purchased pork back fat (C & C Meats, Upper Marlboro, MD) for sausage preparation. Pepperoni chubs were prepared using the multivariate protocols described in Table 1, which are common commercial formulations.

**Table 1:** Curing regimen for production of dry-cured sausage.

% salt 1.3% 1.8% 2.3% 2.8%	% sugar (dextrose) 0.25% (pH 5.2) or 0.7% (pH 4.6)	Nitrate/nitrite [C] (+ 0.05% Na erythorbate) 100/100 ppm	Fermentation temperature/time/pH (Constant temperature process) Fermentation Temp 23.8 °C	Target a_w_ 0.92
	30% fat		Target pH 5.2, 4.6	Drying Temp 15.5 °C RH 75%

Approximately 100 kg of raw pepperoni batter was prepared in each of eight batches. Pepperoni was prepared using *Trichinella* infected pork muscles described above and pork back fat to achieve a ratio of about 70% lean meat to 30% fat. The blend of pork meat and chilled fat was coarse ground using a sanitized mixer/grinder through an accessory grinding plate with 9.5 mm holes (Model 4346; Hobart Corp., Troy, OH, USA), and mixed for 20 min at 4 °C to provide a uniform distribution of parasites. The mixer/grinder was sanitized with a quaternary ammonium-based germicide, multipurpose detergent (Misty® Biodet ND32), rinsed thoroughly for 3 min with hot water (88 °C), and then allowed to air dry at room temperature for up to 1 h. The coarse ground pork was re-loaded into the sanitized mixer/grinder, re-ground to produce to ≥ 3 mm particles, and dry ingredients were added to achieve variable combinations of salt (1.3, 1.8, 2.3, and 2.8% high-grade sodium chloride, The Canadian Salt Company Ltd., Pointe Claire, QC, Canada), 0.25% (for pH endpoint 5.2) or 0.7% (for pH endpoint 4.6) dextrose/sugar to achieve common curing method endpoints for pH (Tate & Lyle of Nealanders International Inc., Mississauga, ON, Canada), and 0.25% cure (sodium nitrate/nitrite; Wiberg Canada Corporation, Oakville, ON, Canada). The pepperoni batter was mixed for about 1 min before the addition of starter culture mixture (*Pediococcus acidilactici* and *Staphylococcus carnosus*) in water (Formula 102; Trumark Inc., Linden, NJ, USA), per the manufacturer's instruction to yield about 6 to 7 log_10_ CFU/g, respectively. The number of ML/g (lpg) of the meat and batter mixtures was determined by digestion of 100 g samples of the final ground meat preparation and each of the eight batter mixtures for isolation of ML and inoculation into mice to determine the initial condition and infectivity of ML as described below. The pepperoni batter was blended for an additional 10 min to assure uniform distribution of additives, finely ground through a 4.7 mm accessory grinding plate, then extruded into water-softened 55 mm diameter cellulose-based casings (Naturin R2; Weinham, Germany) using either a stainless steel table-top manual piston stuffer (9 kgs capacity; Model FD-9051200; F. Dick, Esslingen, Germany) or a floor-type, hydraulic-driven piston stuffer (18.6 kgs capacity; Model SC-50, Koch Equipment, Kansas City, MO, USA), to form pepperoni chubs 254 mm in length, and weighing 500 g each. After stuffing, the pepperoni chubs were hand tied with twine, transferred to a temperature- and humidity-controlled walk-in incubator (EJS Systems Inc., Changrin Falls, OH, USA) with an air flow of 1.0 to 1.5 m/s, and hung vertically on racks so that the individual chubs were not touching. The relative humidity (RH; 88%) and air temperature (23.8 °C) were controlled and constantly monitored during fermentation (and drying at 15.5 °C) using the Dynamist 2000 System (EJS) and Partlow MRC5000 chart recorder (EJS Systems Inc., Changrin Falls, OH, USA). The pepperoni chubs were fermented using constant temperature, degree·hour (the time in hrs at a particular temperature multiplied by the degrees in excess of 15.6 °C (the critical temperature at which Staphylococcal growth effectively begins)), and relative humidity to achieve target pH values of 5.2 and 4.6 as outlined in Table 1. The process used was in compliance with USDA allowances for time at temperatures above 15.6 °C before pH 5.3 was reached (Meat and Poultry Hazards and Controls Guide, 2005). To determine pH during fermentation, at 3 h intervals, 50 g samples from individual chubs representing each treatment were homogenized with 100 ml of deionized water for 1 min in a stomacher blender (Stomacher 3500, Thomas Scientific, Swedesboro, NJ, USA), and the pH of each sample was measured by immersing the electrode of the pH meter in the sample homogenate (Mettler Toledo pH/ion meter, Model 235, Schwerzenbach, Switzerland). Once the targeted pH was achieved (pH 5.2 or 4.6; end of fermentation, 24–28 h at 23.8 °C, 88% RH, a_w_ 0.99–1.0), samples were dried at 15.5 °C, 75% RH, until the target a_w_ endpoint was reached (0.92 a_w_). Water activity and pH in each chub was recorded at the time of sampling. The a_w_ of the samples was measured by placing 20 g samples from individual sausages from each treatment into a sampling cup and analyzing using an electronic water activity meter (Model HP23AW, Rototronic, Hauppauge, NY, USA) calibrated to 80% RH. The pH of sampled chubs was measured as described above.

Four whole chubs from each treatment were randomly removed from the drying chamber and sampled on a daily basis beginning on Day 1 (end of fermentation) through Day 12, then at 2–3 day intervals for digestion to isolate ML for mouse bioassay (described below) to determine infectivity of *Trichinella* ML exposed to conditions of the curing and drying processes. The final samples were collected and digested on Day 42 post stuffing. For digestion, 100 g was collected from each of four chubs, the casing was removed, the meat was chopped by hand into < 3 mm particles, and mixed with 2 l of artificial digestion fluid (1.0% pepsin, w/vol [National Standard Formulary 1:10,000] and 0.37% *v*/v HCl). The ground meat and digestion fluid were mixed vigorously on a magnetic stir plate at 45 °C for 1 h, when there were no undigested pieces of meat visible, then settled for 30 min. The supernatant was decanted, and the sediment was poured through an 80 μm mesh sieve into a round-bottom Pilsner glass. Following settling for another 30 min, the sediment, containing any *T. spiralis* larvae, was washed and settled repeatedly in tap water until the supernatant was clear. The entire sediment, suspended in 50 ml of tap water, was poured into a gridded plastic petri dish (Falcon 1012, Becton Dickinson, Lincoln Park, NJ, USA), and counted using an Olympus stereo microscope at 40 × magnification. Following counting, 500 ML from each digest was orally inoculated into 1 mouse (or the total number of ML, up to a maximum of 500 ML, in 0.5 ml). Forty days after oral inoculation, mice were euthanized by cervical dislocation and thoracotomy, skinned, and eviscerated in accordance with the Guide for Animal Care and Use of Laboratory Animals (BARC IACUC approved protocol # 16-014). Eviscerated and skinned mice were separately digested in total as described above, and the digest sediment examined microscopically for the presence of larvae in the muscle tissue, to determine the continuing presence of viable larvae in the cured pork sample used for the mouse inoculation.

A cut-off of zero live larvae recovered from all four inoculated mice in all successive days (up to 42 days) was the criterion for determining complete inactivation. A *t*-test was used to determine whether the decrease in active larvae between batter and Day 1 was significant. Regressions were used on each pH and NaCl combination to estimate the decrease in active larvae over days.

A cut-off of a_w_ 0.92 on all four chubs was used to determine the end point number of drying days. A linear regression was estimated with pH, NaCl, and their interaction as independent variables and the number of drying days as the dependent variable.

## 3 Results

Upon digestion of 100 g of the freshly ground and mixed meat (pre-cured) selected for preparation of the pepperoni batter, 4667 *T. spiralis* ML (46.7 lpg) were recovered. Two mice were each inoculated with 500 of these recovered ML. Upon digestion after 40 days, approximately 34,000 and 34,750 ML (χ 34,375) were recovered from the two mice, demonstrating the infectivity of the ML present in the initial ground meat preparation used to make the pepperoni batter. Initial water activity (a_w_) and pH of the ground meat was 0.997 and 6.78, respectively.

Pepperoni batter containing the salt, sugar, nitrate, fermentation bacteria, and spice formulations described above was prepared immediately after the meat was ground. Water activity in the mixed batters is described in Table 2; immediately prior to fermentation, the a_w_ of the sausage batters was between a_w_ 0.95 and 0.97. Upon digestion of 100 g of the eight freshly prepared batters, *T. spiralis* ML were recovered from each batter preparation. Pairs of mice were individually inoculated with 500 ML recovered from each of the eight batter preparations. Upon digestion of the mice 40 days later, ML were recovered from each of the two mice from each batter preparation, demonstrating the infectivity of the ML present in the batter preparations used to stuff the casings for pepperoni chubs, in numbers similar to those seen in the original meat preparation (Table 2).

**Table 2:** Numbers of viable ML in mice inoculated with ML from dry cured chubs.

pH, [NaCl]	5.2, 1.3	5.2, 1.8	5.2, 2.3	5.2, 2.8	4.6, 1.3	4.6, 1.8	4.6, 2.3	4.6, 2.8
Meat	34,375^b^/69.5/0.997							
Batter	39,000^b^/78.0/0.976	24,125/48.2/0.977	18,500/37/0.971	32,375/64.75/0.955	38,625/77.25/0.975	44,750/89.5/0.967	18,125/36.25/0.963	32,250/64/0.961
Day 1^a^	1375/2.75/0.975^c^	597.1/1.1/0.973	581/1.16/0.968	347.2/0.69/0.964	162.7/0.32/0.98	812.5/1.62/0.972	500.0/1.00/0.969	812.5/1.62/0.959
Day 2	470/0.94/0.981	587.0/1.17/0.978	85/0.17/0.961	1.2/0.002/0.96	281.2/0.56/0.975	142.0/0.28/0.97	506.0/1.01/0.966	625.0/1.25/0.962
Day 3	702/1.40/0.974	137/0.274/0.968	55/0.11/0.965	73/0.146/0.96	536.7/1.07/0.975	521.0/1.04/0.967	350.0/0.70/0.964	241.5/0.48/0.957
Day 4	250/0.50/0.974	32/0.064/0.968	270.2/0.54/0.96	33.5/0.0670/0.957	29.2/0.058/0.97	250.5/0.50/0.965	584.5/1.17/0.959	163.2/0.32/0.954
Day 5	54/0.11/0.967	0/0/0.964	0/0/0.954	0/0/0.946	200.2/0.40/0.963	327.7/0.65/0.958	20.7/0.04/0.953	38.5/0.07/0.942
Day 6	39/0.078/0.962	80/0.16/0.959	53/0.10/0.953	0/0/0.943	296.7/0.59/0.959	68.7/0.13/0.957	0/0/0.945	0/0/0.941
Day 7	362/0.72/0.969	93/0.186/0.967	33.2/0.06/0.955	0.5/0.001/0.951	1281.2/2.56/0.961	787.5/1.57/0.96	0.25/0.0005/0.947	9.0/0.018/0.938
Day 8	362/0.72/0.971	33/0.066/0.966	9.7/0.019/0.956	0/0/0.945	0/0/0.961	0/0/0.956	0/0/0.943	0/0/0.935
Day 9	100/0.20/0.969	0/0/0.96	0/0/0.955					
Day 10	49/0.098/0.964							
Day 11	0/0/0.965							
Day post stuffing a_w_ 0.92 endpoint reached	38	35	21	12	21	18	12	12

0/0/0 = first day and all subsequent days on which mice were negative for the presence of *T. spiralis* ML.

a *n* = 4 mice.

b *n* = 2 mice.

c χ ML recovered from mice/RCI/a_w_ in meat or chub used for digestion and recovery of inoculated ML.

Pepperoni batters were stuffed into 55 mm casings and fermented for 24–28 h until the final endpoint pH (5.2 or 4.6) was reached. Four chubs from each batter preparation were removed from the fermentation chamber and 100 g from each chub were digested and examined for recovery of ML as described above. Recovered ML (500) were inoculated into four mice to assess infectivity of recovered larvae. After 40 days, viable larvae were recovered from each mouse, demonstrating that some *T. spiralis* ML survived the fermentation process at both pH endpoints (pH 5.2, 4.6) and at all salt concentrations. Water activity in all chubs was 0.959 or greater (Table 2). The number of ML recovered from the mice was significantly lower in mice inoculated with ML from fermented chubs than in mice inoculated with the original meat or batter preparations (all *p <* 0.05; Table 2, Table 3). The number of ML recovered from mice inoculated with ML isolated from chubs immediately after 24–28 h of fermentation was reduced by a minimum of 96% when compared to the number of ML recovered from mice inoculated with ML isolated from batters immediately before fermentation.

**Table 3:** Statistical test results for combinations of two pH and four salt levels.

pH	% NaCl	Test	Estimate	Std.err.	t-Value	p
5.2	1.3	Day 0 vs. day 1	− 3.3451	1.1179	− 2.9922	0.0402
5.2	1.3	Day - linear	− 2.3487	0.3352	− 7.0077	0
5.2	1.3	Day - quadratic	1.1097	0.2195	5.0549	0
5.2	1.8	Day 0 vs. day 1	− 3.6991	1.0708	− 3.4545	0.0259
5.2	1.8	Day - linear	− 2.1371	0.3879	− 5.5093	0
5.2	1.8	Day - quadratic	1.6597	0.3532	4.6993	0.0002
5.2	2.3	Day 0 vs. day 1	− 3.4608	0.4035	− 8.576	0.001
5.2	2.3	Day - linear	− 2.1468	0.571	− 3.7599	0.0017
5.2	2.3	Day - quadratic	1.664	0.5127	3.2457	0.0051
5.2	2.8	Day 0 vs. day 1	− 4.5351	1.3373	− 3.3914	0.0275
5.2	2.8	Day - linear	− 2.5658	2.2312	− 1.15	0.2798
5.2	2.8	Day - quadratic	2.8925	1.5364	1.8826	0.0924
4.6	1.3	Day 0 vs. day 1	− 5.4694	1.3278	− 4.119	0.0146
4.6	1.3	Day - linear	− 1.5108	0.1863	− 8.1081	0
4.6	1.3	Day - quadratic	2.3321	0.3878	6.0129	0
4.6	1.8	Day 0 vs. day 1	− 4.0087	0.1307	− 30.6683	0
4.6	1.8	Day - linear	− 1.5766	0.175	− 9.0075	0
4.6	1.8	Day - quadratic	2.1733	0.2798	7.766	0
4.6	2.3	Day 0 vs. day 1	− 3.5904	0.4747	− 7.5632	0.0016
4.6	2.3	Day - linear	− 1.3681	0.3999	− 3.4214	0.003
4.6	2.3	Day - quadratic	1.4731	0.3382	4.3555	0.0004
4.6	2.8	Day 0 vs. day 1	− 3.3935	0.8678	− 3.9102	0.0297
4.6	2.8	Day - linear	− 2.0137	0.5374	− 3.7469	0.0018
4.6	2.8	Day - quadratic	1.6106	0.3873	4.1583	0.0007

The three estimates are for (1) the difference between day 0 (batter) versus day 1 (approximately 24 h of curing), and tests of the coefficients of (2) linear and (3) quadratic terms in a regression of the number of live larvae over days. The number of live larvae were modeled using a generalized linear model assuming an over-dispersed Poisson distribution; the independent variable for number of days was standardized to mean zero, variance one prior to modeling.

**Fig. 1:**
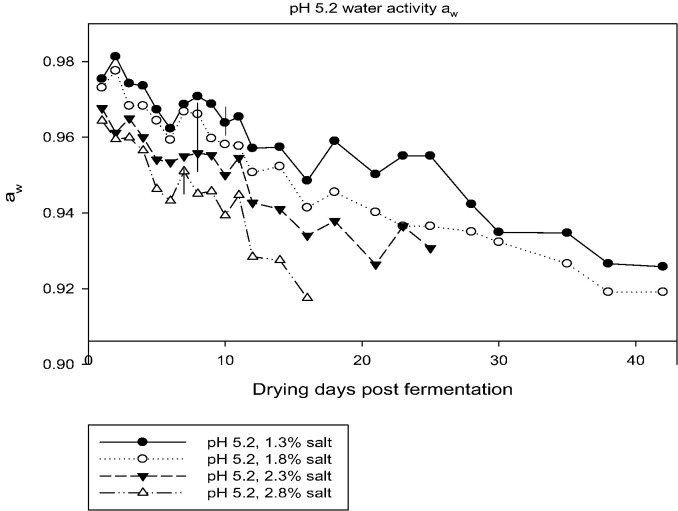
Time in days until sausage chubs reached endpoint a_w_ of 0.92. Vertical line indicates last day upon which infective larvae were found in mice.

**Fig. 2:**
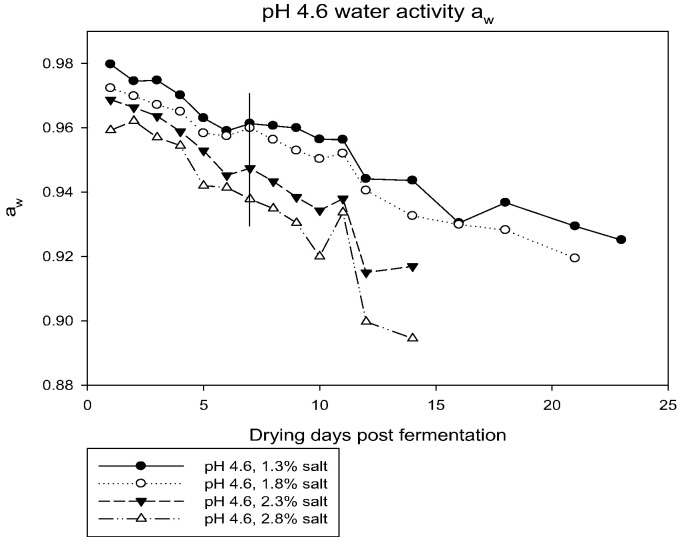
Time in days until sausage chubs reached endpoint a_w_ of 0.92. Vertical line indicates last day upon which infective larvae were found in mice.

Four drying pepperoni chubs from each treatment were collected daily beginning on Day 2 through Day 12, then every 2–3 days until Day 42, and treated as described above before inoculating recovered ML into four mice. After 40 days, ML were recovered from mice by digestion as described above. Results of the ML recovery from mice are shown in Table 2. In each of the chubs prepared from specifically formulated batters, coiled ML were recovered through Day 42. However, when these ML were inoculated into mice, infectivity was significantly decreased over time, by pH (4.6 vs 5.2), and as salt concentrations increased. No ML were recovered from mice inoculated with ML from any pH 5.2 endpoint chub digested after Day 10 (1.3% salt, a_w_ 0.964); Day 8 (1.8% salt, a_w_ 0.966) and (2.3% salt, a_w_ 0.956), or Day 7 (2.8% salt, a_w_ 0.951). No ML were recovered from mice inoculated with ML from any pH 4.6 endpoint chub digested after Day 7 (1.3% salt, a_w_ 0.961), (1.8% salt, a_w_ 0.96), (2.3% salt, a_w_ 0.947), and (2.8% salt, a_w_ 0.938). The pH of each chub was checked at the time of collection. The pH remained stable after fermentation over the course of the 42 day collection period; endpoint 4.6 chubs ranged between pH 4.48 and 4.63, while the endpoint 5.2 chubs ranged between 5.11 and 5.23. Both the main effects of pH and NaCl significantly affected the number of days until a_w_ reached 0.92 (*F*_1,4_ = [32.9, 55.6]; *p* = [0.005, 0.002], respectively, Table 3). During drying of the lower salt preparations (1.3 or 1.8% salt), a_w_ did not reach 0.92 until at least 18 days of drying, and reached a_w_ 0.92 significantly faster in preparations fermented to pH 4.6 than in those fermented to pH 5.2 (test of interaction between pH and NaCl, *F*_1,4_ = 12.4, *p* = 0.024). No ML were recovered from mice inoculated with pepperoni digests where a_w_ reached 0.935 or below, and complete inactivation was achieved in seven of eight batter formulations at a_w_ 0.965–0.943 (Fig. 1, Fig. 2). A summary of the results for complete *T. spiralis* inactivation in dry cured sausage indicates the following proposed rule is applicable:

If pH is 4.6 and % NaCl ≥ 1.3%, 8 days are required to achieve complete inactivation of *T. spiralis* ML.

If pH is > 4.6 and ≤ 5.2, and

if % NaCl ≥ 1.3 but < 1.8, then 11 days are required;

if % NaCl ≥ 1.8 but < 2.8, then 9 days are required;

if % NaCl ≥ 2.8, then 8 days are required.

This proposed rule is not valid for pH outside of the range (4.6, 5.2), nor for % NaCl < 1.3.

## 4 Discussion

*Trichinella spiralis* has presented an intractable problem for producers of RTE meat products containing pork, historically the most common source of the foodborne parasite for humans. Inactivation protocols currently available have involved cooking, freezing, high pressure processing, and salting/curing (Kotula, 1983, Kotula et al., 1983, Smith et al., 1989, Hill et al., 2009, Porto-Fett et al., 2010). However, regulatory requirements for inactivation of *T. spiralis* using methods of curing are limited to only seven validated procedures which are listed in USDA guideline CFR.318.10. The dearth of validated procedures limits producers and regulators in expanding product choices and modifying products to meet changing consumer expectations. An understanding of parameters of meat chemistry which can be used to predict inactivation of *Trichinella* during curing would provide a valuable resource for producers to meet safety requirements. Currently, no general model exists for inactivation of *Trichinella* which is readily accessible to producers and provides validated means to mitigate the risk of their products.

In this study, parameters were identified for inactivation of *Trichinella* using a model curing process; these curing parameters fall within a range of processes which are commonly used by the pork industry for a variety of different dry cured RTE products. Although *Trichinella* viability studies could be (and have been) based on motility or shape of recovered larvae, it is important in food safety studies to demonstrate infectivity of the ML as has been done here (Martinez et al., 1999, Wu et al., 2007, Randazzo and Costamagna, 2010). Not all motile or coiled ML are infective, and some that appear dead can be infective. *Trichinella* larvae were recovered from the mice inoculated with the starting batter with no diminution of infectivity, while the fermentation procedure negatively impacted ML infectivity, reducing recovery of ML by a minimum of 96.5% within 24–28 h. Results indicated that the reproductive capacity (RCI; number of larvae recovered/number of larvae inoculated) of living ML that spent at least 24 h in any of the fermented meat matrices (chubs fermented to pH 5.2 and pH 4.6, 1.3–2.8% NaCl) was negatively impacted by the fermentation process, decreasing from 36.2–89.5 in the batter to 0.32–2.75 on Day 1 after fermentation (Table 2). This rapid inactivation suggests that a_w_ played a minimal role in the inactivation, since the level of unbound water in the chubs did not change over the 24–28 h fermentation process (χ batter a_w_ = 0.968; χ Day 1 a_w_ = 0.97). Additionally, a_w_ remained above 0.95 in six of the eight pH/salt formulations up to the time of complete inactivation of ML (Table 2). Typical endpoint a_w_ for fermented, dry cured sausage such as pepperoni is 0.85 to 0.91 (Getty, 2005).

Since temperatures were held constant at 23.8 °C in all samples during fermentation, the optimal temperature for activity of the fermentation bacteria and typical for the production of a dry cured pepperoni, the impact of the fermentation temperature alone on *Trichinella* infectivity in the batter mixtures could not be determined. Kotula et al. (1983) reported a time and temperature dependent inactivation of *Trichinella* ML; the lowest heating time and temperature combination tested which resulted in inactivation of ML was 49 °C for 6 h. Currently approved *Trichinella* inactivation methods for dry cured sausage without smoking allow drying at temperatures as low as 7.2 °C, however substantial holding times are required (25–65 days) at these temperatures, depending upon the diameter of the sausage (USDA, U.S. Federal Register, 2012). Given that 23.8 °C is below the normal regulated body temperature of typical living mammalian hosts of *Trichinella* ML, and that ML can survive for weeks in muscle of dead hosts at unregulated temperatures, it is unlikely that this temperature alone would result in inactivation.

The difference in pH of 4.6 versus pH 5.2 did impact the number of days on which viable ML were present in chubs; ML did not survive in any case beyond Day 7 in chubs fermented to pH 4.6, while viable ML were present 7, 8, and 10 days after fermentation in chubs fermented to pH 5.2. These data suggest that the rapid drop in pH to 4.6 plays a role in ML inactivation during fermentation as well as during the drying process, in addition to other process variables that were present in the chubs. The pH of pork meat immediately after harvesting from an animal is typically 7–7.2, and may drop to pH 5.5 in < 24 h after slaughter due to formation of lactic acid (Lonergan, 2008). *Trichinella* larvae typically survive for weeks in harvested pork meat, so that a drop in pH to 5.2 alone may not be critical to inactivation of ML, while the drop to pH 4.6 negatively impacts ML survival.

The concentration of NaCl is a significant contributor to ML inactivation. Regardless of pH and water activity, NaCl concentration of 2.8% resulted in loss of infectivity of ML by Day 7. Lower salt concentrations (1.3%, 1.8%, and 2.3%) in conjunction with fermentation to pH 5.2 resulted in the continued presence of infective ML through days 8 and 10. Data from this study indicates that there is a significant interaction between pH and NaCl concentration when modeling the number of days until a_w_ reaches 0.92, demonstrating that these factors are not independent, and that both factors must be considered when developing curing protocols (Table 3).

In conclusion, this study demonstrates that RTE pork sausage, using protocols as described here, can be rendered safe for *Trichinella* infection based upon careful monitoring of pH, salt concentration, and time. One of the largest impediments to trade in pork is the risk or perceived risk of *Trichinella*. Therefore, the results of this study should be valuable in modifying international guidelines (ICT, CODEX, FAO, OIE, etc.) to facilitate trade based on sound scientific data and validated methods. The specific conditions of NaCl concentration, pH and time, as shown in this study, could be used as a baseline to study and/or validate other curing methods, and to begin construction of a generalized model for inactivation of *Trichinella* in RTE pork. The USDA Pathogen Modeling Program (PMP) is a potential vehicle for constructing and facilitating dissemination of such a model. The PMP is a package of models that can be used to predict the growth and inactivation of foodborne bacteria, and are specific to certain bacterial strains and the specific environments (e.g., culture media, food matrix, etc.) that are used to generate the models (Hwang et al., 2009). Since the objectives here were limited to defining relevant parameters, additional data should be generated based on the current findings for development of a complete model of inactivation of *Trichinella*.

## Conflict of interest statement

None of the authors have any financial of personal relationships that could inappropriately influence or bias the content of this paper.
